# Point-of-Care Testing for Aquatic Animal Disease Diagnosis: A Socio-Technical Review of Technologies Enabling Sustainable Aquaculture and Blue Food Security

**DOI:** 10.3390/ani16213518

**Published:** 2026-11-06

**Authors:** Na Jiang, Yukun Zhang, Huanhuan Yu, Xiaoxiao Zhang

**Affiliations:** 1 Fisheries Science Institute, Beijing Academy of Agriculture and Forestry Sciences, Beijing 100068, China; 2 Laboratory of Aquatic Animal Nutrition, Faculty of Fisheries, Kagoshima University, Kagoshima 890-0056, Japan; 3 National Engineering Research Center for Freshwaters, Fisheries Science Institute, Beijing Academy of Agriculture and Forestry Sciences, Beijing 100097, China; 4 Hunan Provincial Key Laboratory of the Traditional Chinese Medicine Agricultural Biogenomics, Changsha Medical University, Changsha 410219, China

**Keywords:** point-of-care testing, aquatic animal health, isothermal amplification, CRISPR diagnostics, antimicrobial resistance, sustainability transitions, sustainable development goals, food security

## Abstract

Disease outbreaks are among the principal constraints on the sustainability of global aquaculture, yet diagnostic capacity remains concentrated in centralized laboratories far from the point of production. Point-of-care testing (POCT)—immunochromatographic strips, isothermal amplification (LAMP, RPA/RAA), CRISPR-Cas assays, microfluidics and biosensors—can compress the diagnosis-to-intervention window from days to about an hour. This structured narrative review draws on a documented search of the Web of Science Core Collection, Scopus, PubMed and the China National Knowledge Infrastructure (2000–2026), with explicit inclusion criteria and a three-tier evidence-grading scheme (Section Review Methodology and Evidence Grading). We synthesize the evidence in the following three tiers: the technical landscape, comparing eight representative platform classes on sensitivity, turnaround, instrument dependence and deployment maturity; the application geography, tracing how POCT has entered pond-side screening, hatchery biosecurity, border inspection of live aquatic trade and IoT-based surveillance networks across East Asian freshwater, Indo-Pacific shrimp and North Atlantic salmonid systems, with markedly uneven evidence of routine adoption; and the bottlenecks that determine whether analytical performance becomes a sustainability outcome—sample-matrix interference, cold-chain dependence, the absence of unified field-performance standards (with WOAH diagnostic-kit registration suspended from May 2024 to May 2027), ambiguous legal status of POCT results, and smallholder affordability. Adopting a sustainability-transition perspective, we argue that the binding constraint is no longer assay chemistry but socio-technical governance, and that POCT should be positioned as enabling infrastructure for antimicrobial stewardship (SDG 3), loss avoidance and trade resilience (SDG 2/12), and aquatic ecosystem protection (SDG 14). We close with coordinated action imperatives for researchers, standard setters, policymakers and industry, including field-validation-first study designs and use of the 2024–2027 standards window to establish graded performance claims for on-site aquatic diagnostics.

## Simple Summary

Diseases in farmed fish and shrimp cause billions of dollars in losses every year, but diagnosing them usually depends on sending samples to distant central laboratories, which wastes critical time. Point-of-care testing (POCT)—easy-to-use tests that work at the pond side, farm, or boat—can return results in about an hour instead of days, letting farmers act quickly. This review examines the main POCT technologies for aquatic-animal diseases, where they are already used around the world, and why many still fail to reach small-scale farmers. We argue that the real bottleneck is no longer the test chemistry but the surrounding rules, standards, and affordability, and we outline practical steps for researchers, governments, and industry to make these tools genuinely useful for sustainable aquaculture and food security.

## 1. Introduction

### 1.1. Global Aquaculture Development and the Worldwide Challenge of Infectious Diseases

Aquaculture is among the world’s fastest-growing food production sectors. According to the State of World Fisheries and Aquaculture [1,2], aquaculture surpassed capture fisheries as the main producer of aquatic animals in 2022; by 2024 it produced 102.7 million tonnes of aquatic animals—53% of the world total, the first time either sector surpassed the 100-million-tonne milestone—and supplied at least one-fifth of animal-protein intake for 3.1 billion people. Growth is driven by major producing economies across continents: East Asia led by China; Southeast Asia (Vietnam, Thailand, and Indonesia) dominating penaeid shrimp and tilapia; Latin America (Ecuador’s whiteleg shrimp, Chilean salmon); and Europe (Norway, Scotland, and the Mediterranean) leading high-value marine finfish. The shift towards high-density, intensive farming has intensified eutrophication, cross-border trade of fry and broodstock, and climate-related stress; against this backdrop, infectious diseases have become the principal biological constraint on the sector and one that is projected to limit future supply rather than merely reduce current output [3].

Table 1 lists the disease of farmed aquatic animals for which a POCT-format assay has been reported, together with a small number of high-impact diseases for which no such assay could be identified.

In penaeid shrimp farming, which is the most disease-constrained sector, white spot syndrome virus (WSSV) remains the single most damaging agent, and acute hepatopancreatic necrosis disease (AHPND), caused by *Vibrio parahaemolyticus* carrying the *pirA/pirB* toxin genes, has been estimated to cost the sector on the order of USD 1 billion annually following its emergence [3,36]. The microsporidian *Enterocytozoon hepatopenaei* (EHP) presents a different diagnostic problem: it is not WOAH-listed, causes chronic growth retardation rather than mortality, and is therefore largely invisible to mortality-based surveillance [6,9,10].

In Asian freshwater aquaculture, the dominant viral threats are grass carp reovirus (GCRV), koi herpesvirus (CyHV-3), spring viraemia of carp virus (SVCV) and megalocytiviruses; WOAH now lists infection with *Megalocytivirus pagrus* 1, which covers red seabream iridovirus (RSIV), infectious spleen and kidney necrosis virus (ISKNV) and turbot reddish body iridovirus (TRBIV) [18]. Tilapia lake virus (TiLV), listed by WOAH since 2021, has spread to most tilapia-producing regions [19,20]. Bacterial disease in the same systems is dominated by *Aeromonas hydrophila*, *Streptococcus agalactiae* and *S. iniae* and *Edwardsiella* spp., each of which now has at least one reported isothermal field assay [21,22,23,24,25,26,27]. In salmonid and marine finfish production, infectious salmon anemia virus (ISAV), infectious hematopoietic necrosis virus (IHNV), viral hemorrhagic septicemia virus (VHSV) and salmonid alphavirus (SAV) remain WOAH-listed concerns [18,28,29,30], while viral nervous necrosis (VNN, betanodavirus) and vibriosis are the principal constraints in marine hatcheries and grow-out [31,32,33].

Disease-related losses are commonly estimated at more than USD 6 billion annually, and the true burden is likely underestimated [37]. Salmon lice (*Lepeophtheirus salmonis*, *Caligus* spp.) alone were estimated to cost the global salmonid industry approximately USD 480 million per year in control costs and lost performance [38]. The experience-based paradigm—visual observation plus empirical medication—proves inadequate across production systems: it delays intervention and drives irrational antimicrobial use, fueling antimicrobial resistance (AMR), as well as raising residue risks and ecological degradation [39,40,41,42]. These are One Health harms: resistant organisms and residues move among farmed animals, aquatic environments and people. Against SDG 2 (blue food security), SDG 3 (health, including AMR), SDG 12 (responsible production) and SDGs 6 and 14 (water and aquatic ecosystems), and in line with the EU Green Deal, China’s green-aquaculture initiative and WOAH’s strategy, on-site rapid diagnostics enabling “early detection, early diagnosis and early intervention” have become indispensable.

### 1.2. Limitations of Traditional Centralized Laboratory Diagnostics

Centralized laboratory diagnosis rests on reference methods prescribed and revised by WOAH in its Manual of Diagnostic Tests for Aquatic Animals [18]. Bacteria are isolated on selective agar and identified by colony morphology, Gram staining and biochemical tests for one to two days for fast-growing vibrios, up to four months for fastidious agents such as Renibacterium salmoninarum, and identification databases are biased towards medically important bacteria [43,44,45]. Viruses are isolated on permissive cell lines (EPC, FHM, and BF-2) and identified by cytopathic effect plus molecular or serological confirmation [18,46]. Histopathology (hematoxylin and eosin, with Gram, immunohistochemical or in situ hybridization staining where indicated) shows agent and host response, but changes are rarely pathogen-specific and light infections are missed [18,47].

Immunoassays (ELISA, IFAT, immunohistochemistry, and slide agglutination) detect antigen or host antibody [18,44,47]. Conventional, nested and real-time quantitative PCR form the backbone of routine confirmation, a positive result normally requiring amplicon sequencing under WOAH case definitions [18,46]. Transmission electron microscopy assigns virions to a family by morphology but cannot resolve within a family and needs a specialist operator [43,46]; wet mounts, bioassay and RFTM culture serve particular host–pathogen combinations [18]. These methods are the reference standard against which any POCT format must be judged [18].

Aquatic health management has long relied on a centralized laboratory system anchored by WOAH reference laboratories and national institutions. These methods deliver analytical accuracy for confirmatory diagnosis but share structural limitations that create a universal gap between laboratory capacity and frontline needs, regardless of national income—summarized in Table 2.

This gap is further amplified by the “sample-to-laboratory” logistics chain. By the time diagnostic results are communicated back to production sites, epizootics have often spread beyond containment, resulting in irreversible losses. These challenges affect both developing nations, where laboratory infrastructure and skilled personnel are scarce, and developed countries with remote farming locations, such as Norwegian fjord salmon farms, Chilean coastal aquaculture sites and isolated inland hatcheries in North America.

Therefore, while traditional laboratory methods remain the reference standard for confirmatory diagnosis, epidemiological surveillance and regulatory testing, they cannot independently fulfill frontline tasks of “early screening, early warning and rapid response” in large-scale, geographically dispersed aquaculture production worldwide. There is an urgent global need for complementary diagnostic paradigms that extend detection capacity closer to the point of production.

### 1.3. POCT Technology and Its Strategic Significance for Global Aquatic Health

Point-of-care testing (POCT) refers to a class of rapid diagnostic technologies that enable sampling, analysis and result reporting at or near the location of the test subject, without dependence on fixed centralized laboratory facilities [51]. Its defining attributes are commonly framed by the WHO criteria spelled out as affordable, sensitive, specific, user-friendly, rapid and robust, equipment-free, and delivered to end users (ASSURED), with short turnaround and minimal instrumentation being especially important in farm settings [51]; these were later extended to REASSURED to incorporate real-time digital connectivity and the capacity to feed surveillance systems, rather than the individual patient alone [52]. POCT has been widely validated across human clinical medicine (blood glucose, blood gas, cardiac markers and infectious disease screening, most notably the global rollout of antigen rapid tests during the COVID-19 pandemic).

Aquatic assays can now be measured against these targets in performance terms, and on speed and sensitivity the reported figures are close. In penaeid shrimp, a commercially validated insulated isothermal PCR assay returned results within 1 h and, over 700 animals of known infection status, 93.5% sensitivity (95% CI 90.6–95.6) and 97% specificity (95% CI 94.3–98.5), at a 95% detection limit of 17 genomic copies per reaction [4]; an RPA–lateral-flow format reached an end point in 30 min at 37 °C, detected 20 copies, and agreed with real-time PCR on all 100 field samples tested [5]; a multiplex RPA–CRISPR/Cas12a–Cas13a assay resolved white spot syndrome virus and Enterocytozoon hepatopenaei in one isothermal reaction readable unaided in under 1 h [6]. Finfish figures are comparable: a non-invasive mucus-swab LAMP test for koi herpesvirus reached 21.4 copies μL^−1^ and full concordance with TaqMan qPCR in 65 min [13]; RPA–CRISPR/Cas12a detected mandarin fish ranavirus and infectious spleen and kidney necrosis virus at 1 and 0.1 copy μL^−1^ within 45 min [17]; and an RT-RPA–Cas12a assay for tilapia lake virus detected 200 copies of RNA at 37–42 °C in under 1 h [19]. No study reviewed here reports a realized per-test price, so affordability remains unevidenced, and this point is taken up in Section 4.

The integration of POCT into fish disease diagnosis and prevention carries multiple strategic values for global aquaculture development and aquatic health governance:

1. Bridging the global diagnostic divide: POCT extends diagnostic capacity to pond-side, hatchery and on-vessel settings, compressing the “sampling—submission—result” cycle from days to roughly an hour, and delivers accessible testing both in resource-limited countries and in remote aquaculture zones of developed economies.
2. Strengthening transboundary biosecurity: POCT provides rapid on-site screening at border inspection posts, import/export ports, quarantine facilities and live-aquatic-animal trading hubs, supporting faster customs clearance and containment of exotic and high-impact pathogens under WOAH international sanitary standards.
3. Advancing sustainable and responsible aquaculture: accurate point-of-care pathogen identification is the diagnostic precondition for targeted and judicious therapeutant use. Whether POCT actually reduces antimicrobial volume in practice is a measured question, not an assumed one, and it is assessed against evidence in Section 5.4.
4. Enabling digital and smart aquaculture globally: POCT devices can be coupled to the Internet of Things (IoT), mobile connectivity, smartphones and cloud platforms, linking with online water-quality monitoring, video surveillance, automated feeding and remote expert consultation to build regional and global intelligent disease-surveillance systems.
5. Supporting equitable aquatic health governance: by lowering the entry barrier to diagnostic capacity, POCT is argued to narrow the health-technology gap between high- and low-income producing nations. Whether that gap has in fact narrowed is examined in Section 6.

Items 3 and 5 are evaluative claims rather than measured outcomes. To avoid restating them, each is substantiated once, in Section 5.4 and Section 6 respectively, and is not re-argued here; Section 2, Section 3 and Section 4 supply the technical and operational evidence on which those assessments rest.

The literature on aquatic POCT is growing but fragmented in four respects. First, performance is reported platform by platform and mostly from developmental or single-site validation, so the technologies have not been compared on a common set of field-relevant criteria: sensitivity and turnaround are routinely reported, whereas instrument dependence, matrix robustness and deployment maturity are not. Second, application evidence is scattered by region, by sector and by position in the production or quarantine chain, and no synthesis shows where POCT has moved beyond demonstration into routine use. Third, the obstacles to translation—matrix interference, cold-chain dependence, the absence of unified field-performance standards, the ambiguous legal status of a POCT result and smallholder affordability—are described in isolation rather than weighed against one another. Fourth, the claims made for POCT beyond diagnosis—antimicrobial stewardship, surveillance reach, and environmental benefit—are asserted more often than they are measured: no included study reports antimicrobial use, mortality, cost or ecological outcomes before and after adoption.

Accordingly, this review of POCT core technologies, applications, bottlenecks and future directions is of direct value for the sustainable development of global aquaculture. The review proceeds from technology landscape (Section 2) through application geography (Section 3) and bottleneck analysis (Section 4) and future trajectories (Section 5); Section 6 converts the assembled evidence into an explicit sustainability assessment, mapping each POCT function onto SDG targets and recording whether the supporting evidence is direct, indirect or absent. As a narrative review, it prioritizes studies with analytical, clinical or field validation and explicitly distinguishes peer-reviewed evidence from institutional, industry and product information wherever adoption data are limited.

### 1.4. Review Methodology and Evidence Grading

This review was conducted as a structured narrative review rather than as a systematic review with meta-analysis. The literature was identified by searching the Web of Science Core Collection, Scopus, PubMed and the China National Knowledge Infrastructure (CNKI) for records published between January 2000 and October 2026; the final search was executed on 20 October 2026. The reference lists of retrieved reviews, and the WOAH Aquatic Animal Health Code and Manual of Diagnostic Tests for Aquatic Animals, were also screened for additional primary sources.

The search combined the following three concept blocks with Boolean operators: (i) technology terms—“point-of-care”, “rapid test”, “lateral flow”, “immunochromatographic”, “LAMP”, “loop-mediated isothermal amplification”, “RPA”, “recombinase polymerase amplification”, “RAA”, “CRISPR”, “Cas12a”, “microfluidic”, “biosensor”, “SERS” and “electrochemical”; (ii) host and system terms—“aquatic”, “aquaculture”, “fish”, “shrimp”, “crustacean”, “mollusc”, “salmonid”, “tilapia” and “carp”; and (iii) target terms—“pathogen”, “disease”, “diagnosis”, “detection” and “surveillance”. Equivalent Chinese-language terms were used in CNKI.

Studies were included if they (i) reported a POCT-format assay—one operable at or near the point of sampling without a central laboratory—for a pathogen or chemical residue relevant to farmed aquatic animals; (ii) provided at least one performance metric (limit of detection, sensitivity, specificity, concordance against a reference method, or turnaround time); and (iii) were published in a peer-reviewed journal or an authoritative institutional source (WOAH, FAO, WHO or national agencies). Studies were excluded if they addressed only human or terrestrial targets without an aquatic application, if they reported food-safety detection lacking an aquatic-pathogen or aquaculture-residue dimension, or if they were conference abstracts, patents or commercial brochures without analytical data. No language restriction was applied beyond the practical English and Chinese coverage of the databases searched.

To separate laboratory proof-of-concept from field validation and from routine deployment, every assay was graded on the same three-tier scheme used in Table 3 as follows: grade A, prospective validation on unselected production samples with diagnostic sensitivity and specificity reported against a reference method; grade B, analytical validation on known-status samples with concordance against an established method in a single laboratory; and grade C, proof-of-concept or methodology work on small, largely spiked or cultured material without a field panel. Routine commercial deployment was recorded separately and accepted only where a product was documented in peer-reviewed or authoritative trade sources; manufacturer claims alone were not treated as evidence of deployment. Because the included studies differ in sample type, reference standard and case mix, performance figures are reported as ranges rather than pooled estimates.

Findings were synthesized narratively by technology class (Section 2), application setting (Section 3) and bottleneck category (Section 4). Where the retrieved evidence was institutional, industry or product information rather than peer-reviewed data, this is stated explicitly at the point of use. The methodological limitations that follow from this design are set out in Section 7.2.

## 2. Core POCT Technologies and Principles for Aquatic Animal Disease Diagnosis

Sustainable aquaculture depends on diagnostic technologies whose performance–deployability trade-off matches resource-constrained farms: the most complex method is not the most sustainable when low-cost, instrument-free, cold-chain-independent alternatives deliver timely, actionable decisions. The POCT landscape rests on three pillars—immunological recognition, nucleic acid amplification and biosensing—organized as a tiered system spanning rough screening, precise confirmation and ultra-sensitive quantification. The comparison below also weighs resource and waste implications (single-use plastics, nanostructured substrates, and electronic readers), because the sustainability case rests on net benefit rather than analytical performance alone.

### 2.1. Immunochromatographic Rapid Detection Technologies

Immunochromatographic technologies are centered on the specific binding of antigen and antibody and rely on lateral-flow systems to achieve instrument-free rapid detection. They are among the most commercialized POCT routes in aquaculture and have a relatively low barrier to adoption at the farm level and comprise the following two main branches: colloidal gold and fluorescent immunochromatography.

#### 2.1.1. Colloidal Gold Immunochromatographic Assay (GICA)

Principle, detection and readout: The strip comprises a sample pad, conjugate pad, nitrocellulose membrane and wick. Colloidal-gold nanoparticles (20–40 nm) label detection antibodies dried on the conjugate pad, while capture and control antibodies form the T and C lines. Capillary flow carries the sample; antigen is sandwiched by the gold-labeled antibody and trapped at the T line as a visible red band, and surplus conjugate is captured at the C line. Read unaided after 10–15 min at room temperature: C plus T lines = positive, C alone = negative, and no C line = invalid. T-line intensity gives only crude semi-quantitative estimates, so the format serves qualitative screening.

Strengths and limitations:

Strengths—no instrumentation, minimal operator training, a long ambient shelf life (validated storage ranges still apply), low per-test cost and tolerance of crude tissue homogenates; a monoclonal-antibody GCRV-II colloidal gold strip detected the virus within 10 min and remained stable at 4–25 °C for 12 months, supporting its suitability for farm conditions [11].

Limitations—sensitivity is only at the ng/mL level, so latent-phase and low-load samples are prone to false negatives; the hook effect weakens the signal at antigen excess; performance depends entirely on antibody affinity and specificity, and antibodies against conserved epitopes may cross-react with related pathogens, while small-molecule targets must be conjugated to carriers; the assay is qualitative and cannot quantify precisely.

#### 2.1.2. Fluorescent Immunochromatographic Assay (FIA)

Principle, detection and readout: The chromatographic architecture matches that of colloidal gold, except that fluorescent microspheres—europium chelates, quantum dots or carboxylated fluorescent latex—are covalently conjugated to the antibody in place of the electrostatically adsorbed gold label, giving lower background and a higher signal-to-noise ratio and commonly improving sensitivity by 1–2 orders of magnitude over GICA, although the realized gain varies with target and system. After the reaction a portable reader measures T- and C-line fluorescence and computes the T/C ratio; against a pre-established, validated standard curve this yields quantitative or semi-quantitative results, and alert thresholds can be set to grade risk rather than force a binary positive/negative call.

Strengths and limitations:

Strengths—sensitivity higher than colloidal gold, a wide linear range and good quantitative accuracy; fluorescence read-out also reduces optical interference from turbid or pigmented matrices relative to visual reading, although autofluorescence and non-specific binding in turbid water or tissue homogenates are not eliminated.

Limitations—a dedicated portable reader is required, so equipment and per-test costs exceed those of colloidal gold, and the fluorescent labels photobleach and need light-protected storage and handling.

### 2.2. Molecular POCT Nucleic Acid Rapid Detection Technologies

Molecular POCT targets nucleic acids, reducing dependence on thermal cyclers through isothermal amplification and combining signal amplification systems to achieve high-sensitivity on-site detection. It is currently the core technology direction for fish pathogen detection and encompasses the following three technology modules: isothermal amplification, CRISPR diagnostics, and microfluidic integration.

#### 2.2.1. Loop-Mediated Isothermal Amplification (LAMP)

Principle, detection and readout: Four core primers (F3, B3, FIP, and BIP) target six conserved regions of the target gene, with optional loop primers (LF, LB) to accelerate the reaction [64]. Through the strand-displacement activity of Bst polymerase, cyclic synthesis proceeds at a constant 60–65 °C and yields cauliflower-like repetitive DNA, reaching ~10^9^ copies within 1 h without thermal cycling [49]. Read-out is by turbidimetry (magnesium pyrophosphate precipitate read by eye or turbidimeter), colorimetry (hydroxynaphthol blue or calcein; the calcein visual scheme is set out by Tomita et al. [50]), lateral-flow dipstick, or gel electrophoresis for laboratory confirmation. Dipstick read-out requires opening the tube, so amplicon aerosol contamination remains a false-positive risk unless closed-format devices are used.

Strengths and limitations:

Strengths—multiple primer recognition regions confer strong specificity, amplification efficiency is high, only a simple constant-temperature device is needed, and several formats allow naked-eye read-out. LAMP was also the earliest isothermal technique applied to aquatic pathogens, with RT-LAMP for viral hemorrhagic septicemia virus [29], RT-LAMP-LFD for grass carp reovirus [12], visual LAMP for koi herpesvirus [13] and an early review of fish and shellfish applications [48].

Limitations—primer design is complex and requires dedicated software, the 60–65 °C requirement constrains the heating device, open-tube read-out carries aerosol contamination risk, and efficiency declines for long amplicons.

#### 2.2.2. Recombinase Polymerase Amplification (RPA/RAA)

Principle, detection and readout: RPA, proposed by Piepenburg et al. [65], uses a recombinase (T4 uvsX), single-stranded DNA-binding protein (gp32) and a strand-displacing polymerase (Bsu large fragment): the recombinase–primer complex scans for homology and mediates strand invasion, and the polymerase extends from the primer 3′ end, giving exponential amplification from fewer than 10 copies at 37–42 °C within 15–30 min under the reported conditions [65]; lyophilized formats permit ambient-temperature transport, though stability depends on formulation and storage [61]. Read-out is by exo-probe fluorescence (a dual-labeled probe cleaved during amplification and monitored in real time on a portable fluorometer), nfo-probe lateral-flow dipstick (biotin plus hapten labels captured on a strip for instrument-free reading, as Piepenburg et al. [65] themselves demonstrated), or agarose gel electrophoresis for laboratory verification.

Strengths and limitations:

Strengths—a reaction temperature close to body temperature requiring only a very low-power heater, completion within 20–30 min [65], lyophilizable reagents that escape cold-chain constraints, and strong tolerance of crudely processed samples; in aquaculture, lateral-flow RPA has been used for Enterocytozoon hepatopenaei [9] and RPA–CRISPR/Cas12a for field detection of acute hepatopancreatic necrosis disease [7].

Limitations—primer design is more demanding than for LAMP, with strict requirements on length, GC content and secondary structure; inhibitor tolerance is limited, so debris-rich samples need simple purification; reagent cost exceeds that of LAMP; efficiency falls sharply once the amplicon exceeds ~500 bp; and dipstick read-out carries the same open-tube contamination risk.

#### 2.2.3. CRISPR-Cas12a Ultra-Sensitive Detection Technology

Principle, detection and readout: Detection exploits collateral cleavage—the Cas12a–crRNA complex recognizes target DNA adjacent to a TTTV PAM and then cuts free single-stranded DNA indiscriminately [66]. Chen et al. [66] coupled RPA pre-amplification to Cas12a cleavage in DETECTR, reaching attomolar detection and HPV16/18 genotyping within 1 h at 37–42 °C, with recognition and cleavage in 10–30 min. Read-out is by fluorophore–quencher reporter probes (portable fluorometer, or by eye under blue light [66]), colloidal gold strips, or gold-nanoparticle colorimetry. In the mainstream positive-band strip format an intact biotin–FAM reporter is retained at the C line; once cleaved, the FAM-bearing fragment is carried to the upstream T line by gold-conjugated anti-FAM antibody, so the C line weakens in strongly positive samples and signal-decreasing formats must be distinguished from signal-increasing ones.

Strengths and limitations:

Strengths—very high specificity, with appropriately positioned mismatches (particularly in the PAM-proximal seed region) discriminating closely related genotypes and, in optimized formats, single-nucleotide differences [67]; attomolar sensitivity once coupled to pre-amplification [66]; strong signal amplification with high-contrast results; and gene-level resolution combined with isothermal portability. In aquaculture, an RPA–CRISPR/Cas12a system achieved on-site detection of AHPND in shrimp within 2 h at an analytical limit of 200 copies/µL, with 100% negative and 92.5% positive percent agreement against field samples [7].

Limitations—target selection is constrained by PAM availability, peak sensitivity requires pre-amplification, system components are complex and relatively costly, and collateral cleavage is sensitive to environmental impurities and reaction pH.

#### 2.2.4. Microfluidic Integrated POCT Technology

Principle, detection and readout: Micro-electromechanical fabrication places microchannels, microvalves, micropumps and reaction chambers on centimeter-scale substrates (polydimethylsiloxane, glass, and paper), closing the entire workflow—sample pre-treatment, nucleic acid extraction and purification, isothermal amplification or CRISPR reaction, and signal detection—within a single chip and removing manual transfer. Paper-based devices patterned by hydrophobic wax printing drive flow by capillary force with no external power, whereas polymer chips use precision pumps and valves for more complex multi-step reactions. Read-out uses integrated fluorescence windows, electrochemical electrodes or colorimetric zones read by a dedicated portable instrument; some schemes instead photograph the reaction with a smartphone camera and apply algorithms for automated determination and cloud upload.

Aquaculture demonstrations include: an integrated microfluidic system that completes magnetic-bead extraction, LAMP amplification and optical detection within 65 min (limit of detection 20 copies; four parallel modules for *S. agalactiae*, koi herpesvirus, iridovirus and *A. hydrophila*) [21]; an amplification-free magnetic-bead/molecular-beacon system detecting nervous necrosis virus and iridovirus within 30 min [31]; and a dual-sample LAMP chip detecting ten aquatic pathogens simultaneously in under 30 min (reporting 93.52% sensitivity and 85.53% specificity against laboratory-confirmed isolates, not a clinical or regulatory evaluation) [22].

Strengths and limitations:

Strengths—a fully closed workflow greatly reduces aerosol contamination; sample and reagent consumption falls to the microliter scale; automation is high and operating steps few, limiting human error; and multi-target parallel detection is achievable [22].

Limitations—chip fabrication is complex and mass-production costly; microchannels clog on tissue homogenates and extraction from debris-rich samples is inefficient; and most aquaculture reports remain laboratory proof-of-concept with few commercial products, consistent with cross-domain analyses showing that only a small fraction of microfluidic diagnostics reach market [63] and with aquaculture-specific reviews reporting negligible field deployment [58].

### 2.3. Emerging Biosensing and Nano-POCT Technologies

Biosensing technologies couple biorecognition elements with signal transducers, converting pathogen-recognition events into quantifiable physical signals; many formats dispense with the separation or washing steps required by conventional immunoassays and respond within minutes. This makes them an important technological route for realizing integrated “instant testing—data acquisition”.

#### 2.3.1. Electrochemical Biosensors

Principle, detection and readout: Disposable screen-printed electrodes carry immobilized biorecognition elements (antibodies, aptamers, and molecularly imprinted polymers); target binding alters electron transfer at the interface, shifting current, voltage or impedance, read by differential pulse voltammetry, electrochemical impedance spectroscopy or amperometry [68]. A USB-sized portable potentiostat reads the signal and converts it to concentration through a standard curve, and some devices link to a phone by Bluetooth for in-app display. Aquaculture demonstrations include an aptamer sensor for *Aeromonas hydrophila* and *Edwardsiella tarda* reporting ~1 CFU/mL in spiked or optimized samples without culture—an assay-level estimate, not a field-validated sensitivity [23]—and a COF/MWCNTs/chitosan enrofloxacin aptasensor with a miniaturized device for “sample-in—result-out” residue testing in real water [69].

Strengths and limitations:

Strengths—high sensitivity; a small, low-cost, easily carried device; fast response, mostly within 10–30 min; and easy arraying for simultaneous multi-analyte detection.

Limitations—batch-to-batch reproducibility of electrode modification is poor, compromising result consistency; matrix interference from fish tissue homogenates and aquaculture water is strong and non-specific adsorption occurs readily; and immobilization of recognition elements is a multi-step, process-intensive procedure whose reagents have a relatively short shelf life.

#### 2.3.2. Surface-Enhanced Raman Scattering (SERS) Sensing

Principle, detection and readout: Localized surface plasmon resonance on gold or silver nanostructures enhances the Raman signal of adsorbed molecules (ensemble enhancement 10^6^–10^10^, hot spots 10^14^–10^15^ [70]); immunocapture, aptamer capture or magnetic enrichment concentrates the target at the interface so that a fingerprint spectrum can be acquired. A portable Raman spectrometer then identifies the target by peak position and quantifies it by peak intensity, with chemometric or machine-learning support for multi-component recognition. Aquaculture demonstrations include label-free SERS combining Fe_3_O_4_@PEI magnetic enrichment with Au@Ag core–shell nanoparticles to detect five bacterial pathogens (including *V. anguillarum*, *V. harveyi* and *E. tarda*) within 20 min with linear-discriminant classification [27]; a trifunctional edged-satellite AuAg platform covering *V. parahaemolyticus*, *V. vulnificus* and *V. alginolyticus* (2 min colorimetric screen, SERS confirmation at ~50 CFU/mL, and in situ sterilization) [34]; and a nanoaggregate-embedded-bead multiplex assay for the pirA/pirB/ompA genes of *V. parahaemolyticus* down to ~10^−16^ M [35].

Strengths and limitations:

Strengths—fingerprint-level specificity that distinguishes structural analogs, potentially high sensitivity, multiplex detection and simple pre-treatment.

Limitations—poor substrate uniformity and measurement reproducibility, strong background from complex matrices, costly portable Raman instruments, and demanding substrate storage. Objectively, “single-molecule level” mostly reflects ideal substrates and pure cultures: in aquaculture water and tissue homogenates, published bacterial limits of detection lie mostly at 10^2^–10^5^ CFU/mL, with only a few optimized platforms reaching ≤10^2^ CFU/mL.

#### 2.3.3. Other Nanobiotechnology Platforms

- Quantum dot FRET sensing: quantum dots act as fluorescence donors and organic dyes as acceptors; the target alters the donor–acceptor distance or displaces a competitor, switching on FRET and producing donor quenching with acceptor-sensitized emission. Strengths are photobleaching resistance, high quantum yield and multicolour multiplexing; limitations are the potential biotoxicity of quantum dots and the complexity of surface modification.
- Upconversion nanoparticle luminescent sensing: rare-earth-doped nanoparticles emit visible light under near-infrared excitation (anti-Stokes emission), giving no autofluorescence background, a very high signal-to-noise ratio and good photostability; the penetration depth of near-infrared excitation matters mainly for tissue imaging and is of limited relevance to in vitro aquatic diagnostics. Limitations are low luminescence efficiency and difficult synthesis and modification.
- Molecularly imprinted polymer biomimetic sensing: polymers are cast around the target molecule and, after elution, leave cavities complementary to its spatial structure and functional groups, substituting for natural antibodies. Strengths are stability to acid, alkali and heat, low cost and easy batch preparation; the limitation is lower recognition specificity for macromolecular pathogens than natural antibodies. Nanozyme-catalyzed color development is increasingly coupled with such artificial recognition elements, although reported applications to date come from food and water matrices rather than aquaculture [71].

Note: the three directions above are frontier, exploratory technologies; published aquatic-pathogen studies remain largely proof-of-concept, and mature products appear to be scarce. The broader nanomaterial-enhanced lateral-flow landscape in aquaculture is reviewed by Jaies et al. [72], upconversion-nanoparticle lateral-flow platforms by He et al. [73]; smartphone-coupled readouts include RPA–CRISPR/Cas12a microscopy for frog virus 3 [74] and a 3D-printed multiplex LAMP chip for six targets [54]—illustrating the trend towards digital, instrument-light diagnosis.

### 2.4. Comprehensive Performance Comparison of Mainstream POCT Technologies

The technologies surveyed in Section 2.1, Section 2.2 and Section 2.3 are compared across analytical performance and deployment characteristics in Table 3.

The various technologies differ in their targets, sensitivity, equipment needs, and operational difficulty; rather than direct substitutes, they form a complementary, tiered technology system. Overall, immunochromatography is suited to large-scale, low-cost primary screening; isothermal amplification and CRISPR technologies can support high-specificity on-site confirmation; and biosensing and microfluidic technologies are evolving toward integration, quantification and intelligent networking.

**Table 3:** Comparative characteristics of principal POCT technology platforms for aquatic-animal disease diagnosis.

Technology Class	Sub-Technology	Target	Reaction Time	Limit of Detection	Equipment Needs	Operational Difficulty	Quantification	Maturity	Core Advantage	Main Shortcoming	Evidence Quality #	Validation Sample Size	Matrix-Specific Limitations	Commercial Availability	Field Readiness	Representative References in Aquaculture
Immunochromatography	Colloidal gold GICA	Protein/antigen/whole cell	10–15 min	ng/mL level	None	Very low	Qualitative/semi-quantitative	High (commercialized products available)	Zero instruments, low cost, simple operation	Low sensitivity, hook effect	B/C—kits validated mainly on known-status or spiked material	Tens of samples per study, mostly known-status or spiked	turbid or pigmented tissue homogenates and hemolymph impede strip flow and visual scoring	High—WSSV; AHPND PirAB toxins [8]; GCRV [11]; NNV [33];	High—no instrument, ambient storage, 10–15 min, visual read-out, non-specialists	[8,11,33,75]
Immunochromatography	Fluorescent FIA	Protein/antigen/whole cell	10–15 min	pg/mL–ng/mL level	Portable fluorescent reader	Low	Semi-quantitative/quantitative	Medium-high	Relatively high sensitivity, quantitative	Requires paired instrument, slightly higher cost	C—read-out transferred from food and medical multiplex platform; aquaculture field panels remain scarce	Validation rests on spiked panels and replicate measurements	Needs a clarified extract; sample autofluorescence and turbidity raise background	Moderate—aquaculture-specific quantitative panels remain limited	Moderate—portable reader, but needs power, calibration and reagent cold chain; not a stand-alone pond-side test	[75,76]
Isothermal amplification	LAMP	Nucleic acid	30–60 min	10–10^2^ copies per reaction	Simple constant-temperature device	Low–medium	Qualitative/semi-quantitative	Medium-high (rich aquaculture methodology)	High amplification efficiency, diverse detection formats	Challenging primer design; open-lid readout poses a risk of aerosol contamination	B—the best-documented isothermal format in aquaculture	Tens to roughly 100 samples per study	Tolerates crude extracts better than PCR, but humic substances inhibit fluorescence-based read-outs	Moderate—kits are sold for several shrimp and fish pathogens, but mostly as laboratory kits	Moderate–high—single heater at 60–65 °C, 30–60 min, visual colorimetric or turbidity read-out	[13,49,50]
Isothermal amplification	RPA/RAA	Nucleic acid	15–30 min	<10 copies (system level)	Simple constant-temperature device	Low	Semi-quantitative/quantitative	Medium	Low temperature, fast, lyophilizable reagents	Challenging primer design and higher cost; open-lid readout poses a risk of aerosol contamination	A/B—the strongest field evidence of any isothermal class	Up to 100 field samples in the best-documented assay	Lyophilized reagents and strip read-out tolerate crude extracts, open-tube handling still risks carry-over	Good—commercial recombinase kits and lyophilizable reagent formulations available	High—single low temperature, 15–30 min, lyophilizable reagents and strip read-out	[9,10,65]
CRISPR diagnostics	Cas12a (with pre-amplification)	Nucleic acid	40 min–2 h (incl. pre-amplification and readout)	aM level	Constant-temperature device + fluorometer/test strip	Medium	High quantification potential	Medium (aquaculture mostly research-stage)	High sequence specificity; genotype resolution depends on assay design	Complex system, high cost, PAM constraint	C/B—mostly proof-of-concept; no prospective field panel reported	mostly reference material rather than unselected production samples	Requires pre-amplification, multiple transfer steps raise contamination risk	Low—no licensed aquaculture CRISPR assay	Low–moderate—laboratory prototypes dominate; one-pot and lyophilized formats are emerging but still need equipment	[6,16,66,67]
Microfluidics	Integrated chip	Nucleic acid/antigen	30–65 min	Depends on core reaction (aquaculture demonstration: 20 copies)	Portable reader	Very low	Quantitative	Medium-low (R&D stage)	Can be fully closed and automated, with multi-target parallelization	Chip cost and complex-sample compatibility can be limiting	C—integrated chips demonstrated on cultured or spiked material; no prospective production-sample validation	Usually <30 per study; these are engineering demonstrations rather than diagnostic cohorts	Particulates clog channels and foul surfaces; on-chip pre-treatment is still required and complex-sample compatibility is a limitation	Low—commercialization of point-of-care microfluidics has proven slow even in higher-value human markets	Low—portable reader required, chip cost and fabrication quality control unresolved; not field-hardened	[21,22,31,63]
Biosensing	Electrochemical sensing	Antigen/small molecule/toxin	10–30 min	pg/mL level, CFU/mL level	Portable potentiostat	Low–medium	Quantitative	Medium	Compact device, high sensitivity, fast response	Reproducibility and matrix interference can be challenging	C—benchtop analytical validation, largely in buffer or simple matrices; aquaculture demonstrations cover enrofloxacin residues and fish-pathogen aptasensing	Tens of measurements, generally replicate calibrations rather than independently sampled animals	Electrode fouling and matrix interference; ionic strength and pH shift the signal and calibration drifts between runs	Low–moderate—portable potentiostats and screen-printed electrodes are sold, but no validated aquaculture assay is marketed	Low–moderate—compact and fast, but needs calibration, a stable reference electrode and clean sample handling	[23,68,69]
Biosensing	SERS sensing	Whole cell/toxin/drug	10–30 min	Reported mostly 10^2^–10^5^ CFU/mL in real matrices, depending on substrate and assay	Portable Raman spectrometer	Medium	Quantitative	Low–medium	Fingerprint identification, multi-component simultaneous detection	Poor substrate uniformity, expensive instrument	C—proof-of-concept on cultured bacteria and spiked matrices, 10^2^–10^5^ CFU/mL in real matrices	Reported as replicate spectra rather than animal panels	Strong background from pigments, organic matter and salts; substrate fouling	Low—substrates are largely laboratory-made; portable Raman instruments exist but are costly	Low—requires a Raman spectrometer with optical alignment and controlled lighting and vibration	[27,34,35,70]

Note: Reaction times refer to complete workflows where stated; Cas-only cleavage steps typically take 10–30 min. “Maturity” denotes aquaculture-specific readiness: High = commercial products available; Medium = validated methods with limited commercial availability; Low = laboratory prototypes. # Evidence quality is graded consistently across rows: A = prospective validation on unselected production samples with diagnostic sensitivity and specificity reported against a reference method; B = analytical validation on known-status samples with concordance against an established method, single laboratory; C = proof-of-concept or methodology study on small, largely spiked or cultured material, with no field panel. Sample sizes are reproduced as reported by each study and are not pooled, because the studies differ in sample type, reference standard and case mix.

## 3. Application Status of POCT Technologies in Global Aquatic Animal Disease Control

Field deployment does not track technical maturity: commercial readiness is highest where industrial production is most concentrated, while the regions with the heaviest disease burdens still depend on the least validated tools. Applications are organized into three tiers—farm-level “early warning” and on-site testing (Section 3.1), inspection and quarantine in international trade (Section 3.2), and networked, data-linked deployment (Section 3.3)—followed by an illustrative worldwide synthesis (Section 3.4).

### 3.1. “Early Warning” and On-Site Rapid Screening at Farming Sites

The production frontline is both the most central and the most demand-driven application scenario for fish disease POCT, and its value is concentrated at the following two critical nodes: rapid confirmation and targeted intervention during the early phase of disease onset, and up-front risk control at seed (juvenile) introduction. Around the world, the realization of this value takes different forms and follows different technological pathways depending on the level of development of each production region.

#### 3.1.1. On-Site Self-Testing and Precise Intervention During Early Disease Onset

The progression window of fish diseases is extremely short—often only 2–3 days from non-specific signs to mass mortality in high-density systems. Under the traditional model, farmers pre-emptively disinfect or medicate on experience alone, and laboratory results arrive after the optimal intervention window. POCT reconstructs the chain as anomaly detected → on-site sampling (moribund tissue, culture water) → lateral-flow or isothermal result in 15–60 min → targeted intervention—turning “speed” from a convenience into a core component of control efficacy.

- East Asian freshwater aquaculture (grass carp and other Chinese major carps) is the most active region for POCT methods research. A colloidal-gold strip targeting the VP35 capsid protein of grass carp reovirus type II (GCRV-II), developed with a double-antibody sandwich format, completes detection within 10 min (against which the 1.76 × 10^2^ copies/µL viral load was determined by qPCR rather than claimed as the strip’s own antigenic LOD), remains stable for 12 months at 4–25 °C, and matches the national reference method (GB/T 36190-2018) [11]; an RT-LAMP–lateral-flow dipstick assay for GCRV genotypes I/II completes amplification and readout within 50 min at 63 °C, 1–2 orders of magnitude more sensitive than conventional RT-PCR [12]. Commercial colloidal-gold strips for banned drug residues (malachite green, chloramphenicol) are in grassroots use (e.g., Shenzhen IRIS, Wuhan Yupinyan; the manufacturer literature), whereas standardized POCT for bacterial pathogens such as *Aeromonas hydrophila* and *Edwardsiella* spp. remains limited. Public evidence is still dominated by method development rather than systematic surveys of routine, scaled operation by grassroots farmers, cooperatives, or county-level aquatic animal health agencies—a clear research gap.
- Southeast Asian and Latin American shrimp farming has reported comparatively advanced commercial POCT deployment. WSSV and AHPND are the two most damaging regional pathogens; AHPND-associated losses accumulated in Asian penaeid shrimp over 2010–2016 at an industry-estimated ~USD 44 billion (a cumulative, region-specific figure not directly comparable with the >USD 6 billion annual global all-pathogen benchmark cited in Section 1.1) [7,36]. Methodologically, an RPA–CRISPR/Cas12a lateral-flow assay for the AHPND virulence genes pirVPA/pirVPB completes detection within 2 h (limit of detection 200 copies/µL; field PPA 92.5%, NPA 100%) [7]; insulated isothermal PCR (POCKIT™) devices are validated for shrimp pathogens [4,55]; RPA–lateral-flow dipsticks target EHP [9,10]; and DNA lateral-flow strips for WSSV/AHPND reach 92.6% sensitivity and 92.9% specificity on infected tissues [77]. Commercially, the Forte Biotech RAPID platform operates in nine countries across six shrimp pathogens including WSSV, AHPND, EHP, TPD and IMNV [78], and a polyclonal-antibody PirAB LFIA validated on Vietnamese farms achieves 94.0%/98.0% sensitivity/specificity [8]. Duplex PCR remains the laboratory comparator for distinguishing classical from mutant AHPND [79].
- European and South American Atlantic salmon farming is dominated by industrial cage aquaculture. Since ISA first appeared in Norway in 1984, annual confirmed outbreaks have been held to roughly 1–25 through a rigorous surveillance and biosecurity system [28]. Principal viral threats include ISAV, IHNV, salmonid alphavirus/pancreas disease virus (SAV/PDV), infectious pancreatic necrosis virus (IPNV), piscine myocarditis virus (PMCV; cardiomyopathy syndrome) and piscine orthoreovirus (PRV; heart and skeletal muscle inflammation); IHNV is WOAH-listed, with Chile treating IHNV exclusion as a priority in salmonid egg-import quarantine. The Norwegian Veterinary Institute (a WOAH Reference Laboratory) applies RT-PCR, qPCR, ddPCR and virus isolation as routine methods, with IFAT for confirmatory isolate identification [28,80]; ISA-restricted zones require monthly farm inspections with results aggregated by the Norwegian Food Safety Authority. Isothermal-amplification rapid testing (iiPCR, LAMP) for salmonid pathogens has been reported [81] and addresses a genuine need to shorten sea-site sample turnaround but remains exploratory: routine deployment is not yet documented.

For global farming sites where mixed and secondary infections are the norm, the speed dimension itself is what makes POCT irreplaceable for control efficacy relative to conventional laboratory methods.

#### 3.1.2. Risk Control at Seed Introduction and in Routine Inspection

Cross-border movement of seed stock is an important route for disease spread. Pre-stocking quarantine screening with on-site rapid tests can intercept virus-positive seed before release, preventing a “Trojan-horse” introduction that infects an entire farm; in mid- to late culture, combining POCT with online water-quality monitoring and pond patrols creates a dual-indicator early-warning mechanism (“water-quality anomaly + pathogen rapid test”) that materially extends outbreak lead time.

From a global practice perspective, POCT use is markedly more mature in large-scale hatchery and breeding systems than on the smallholder farming side. Scaled breeding systems such as Norwegian salmon broodstock facilities, Thai shrimp broodstock farms, and China’s national-level aquatic germplasm conservation and breeding farms commonly operate three sampling checkpoints—“testing at entry, testing during spawning and rearing, testing at exit” (industry practice; systematic documentation remains limited). However, RPA/LAMP isothermal amplification combined with lyophilized reagents and lateral-flow strips is still at an exploratory stage, and POCT penetration remains low.

### 3.2. Inspection and Quarantine in International Trade of Aquatic Products and Seed

Customs ports of entry and aquatic broodstock farms are the “national gateway” and the “germplasm gateway” of global fish disease control. The World Organization for Animal Health (WOAH, formerly OIE) Aquatic Animal Health Code lists several dozen fish and crustacean diseases as priority controls for cross-border trade, and the major trading countries have each established tiered quarantine regimes. Within this global regulatory framework, the application value of POCT manifests at three levels.

#### 3.2.1. On-Site Initial Screening at Border Ports of Entry

For imported live aquatic animals such as ornamental fish, eggs, live fish, and broodstock shrimp, colloidal gold strips and portable RPA/LAMP devices can complete rapid initial screening for WOAH-listed diseases at port quarantine facilities, minimizing both detention time and transmission risk during the “waiting for laboratory confirmation” period. Several typical directions are already supported by public evidence:

1. Ornamental fish trade: in the global koi and ornamental fish trade chain, field-applicable rapid tests for cyprinid herpesvirus 3 (CyHV-3/KHV) have moved from concept to practice: a PCR–lateral-flow point-of-care system for CyHV-3 passed diagnostic validation for field use [14], a pond-side test was used through a full-season koi herpesvirus outbreak survey in the United Kingdom [15], and a non-invasive mucus-swab visual LAMP point-of-care assay for KHV achieved a detection limit of 21.42 copies/µL with 100% concordance against TaqMan qPCR [13]. These tools make a two-tier “rapid screening—laboratory confirmation” workflow at major trade nodes technically feasible, although systematic evidence of its formal adoption at EU, Japanese, or Chinese ports remains to be documented.
2. Shrimp export trade: on-site RPA–lateral-flow assays for WSSV and RPA–CRISPR/Cas12a assays for AHPND have been validated with shrimp-farm samples [5,7], and industry reporting documents commercial rapid on-site diagnostic tools being deployed for AHPND management in export-oriented production [82]. Their systematic use as a pre-shipment internal quality-control procedure in Thailand, Vietnam, Indonesia, and Ecuador is an industry practice described in the trade literature rather than in peer-reviewed surveys and importing countries’ ports increasingly view such tools as candidates for supplementary spot verification.
3. Official border inspection tools: at official entry inspections, conducted for example at EU border control posts (BCPs) and by the U.S. Animal and Plant Health Inspection Service (APHIS), the potential of POCT as a risk-stratification and sample-triage tool has been widely discussed, but explicit regulatory provisions designating it as a “priority initial screening tool for high-risk consignments” have not yet been codified in publicly available legislation, and this remains a direction being gradually explored in national regulatory practice.

#### 3.2.2. Continuous Surveillance at Broodstock Introduction Quarantine Facilities

Global breeding introductions generally follow three-stage management: overseas quarantine → entry isolation → local acclimatization. Periodic POCT sampling of batch specimens at quarantine and broodstock facilities builds a full-chain biosecurity barrier against introduction and vertical transmission. For salmon, shrimp and tilapia, large breeding operations report POCT screening combined with laboratory confirmation, but systematic surveys are lacking and smallholder penetration remains constrained by cost and training.

#### 3.2.3. Disease Traceability and Emergency Response

A positive rapid-test result at a port of entry can trigger emergency measures—culling, biosecure disposal, zone lockdown, and trade suspension—with laboratory confirmation completing statutory procedures: a two-tier “rapid screening first, confirmation as backstop” architecture balancing timeliness and legal validity. Such measures have been reported in SVCV, WSSV and ISAV emergency responses, though documented cases remain scattered. Notably, the WOAH Aquatic Animal Health Code prescribes laboratory-based procedures for official certification and, to our knowledge, does not appear to specify a pathway under which POCT results alone constitute admissible statutory evidence; POCT functions as a risk signal triggering confirmatory testing, and its legal status in cross-border regulation awaits international standards and national legislation.

### 3.3. Convergence with Smart Fisheries and Global Aquatic Animal Health Surveillance Networks

The value of POCT as a single-point test is limited; once results are digitized and networked, its value leaps from “one test per pond” to “regional epidemic situational awareness.” This convergence with smart fisheries thus represents a key development direction for global aquatic disease control.

#### 3.3.1. Internet-of-Things Enablement of Testing Devices (IoT-POCT)

Portable fluorescence strip readers and isothermal amplification devices fitted with 4G/5G or Bluetooth modules can automatically upload the detected pathogen, testing time, GPS coordinates, and farm information to a cloud platform upon completion of the assay, without manual data entry, thereby reducing data latency and the scope for human error or manipulation at the point of data generation. This model is being explored in several scaled production regions:

1. Norway, as a major Atlantic salmon producer, has promoted automated farm-data collection through industry-wide data-sharing initiatives such as AquaCloud, but the current public literature focuses mainly on water quality, feeding, biomass, and camera-derived health indicators—reviews of data use for salmonid health management and of aquaculture-IoT applications confirm this production-parameter emphasis [83,84], and Norway’s BarentsWatch Fish Health platform likewise aggregates observer and laboratory data rather than device-generated POCT results; systematic integration of aquatic animal disease rapid-test data with the platform remains at an exploratory stage;
2. China’s Ministry of Agriculture and Rural Affairs issues an annual national aquatic-animal disease monitoring plan (e.g., MARA [85], the 2024 National Aquatic Animal Disease Monitoring Program), which underpins provincial reporting systems; sector associations have also convened national workshops on optimizing aquatic-animal disease remote-diagnosis and early-warning cloud platforms [86]. Pilot connections between field rapid-test data and fishery management cloud platforms are therefore documented in policy and industry sources, though peer-reviewed evaluations of their coverage and effectiveness remain absent;
3. Large shrimp farming groups in Thailand and Ecuador have reported enterprise-level unified health-management practices involving IoT-enabled testing devices, but systematic effectiveness evaluations in the public academic literature remain scarce.

What remains at the pilot or proposal stage is the automatic upload of device-generated POCT results into these platforms and their fusion with environmental and production streams. Because these links are recent and rest largely on vendor and industry documentation, independent evaluations of their data completeness, interoperability with farm-management and surveillance systems, accuracy under networked operation, and measurable effect on surveillance outcomes are not available.

#### 3.3.2. Mobile-Terminal Operation and Intelligent Readout

A smartphone can serve as both reading terminal (photometric or fluorescence apps) and data-entry point; AI image recognition of T/C lines reduces the subjectivity of naked-eye readout, making “one phone + one strip” the lowest-cost digital testing unit. In infrastructure-poor regions this substantially lowers the POCT threshold and, combined with mobile testing vehicles, reaches remote small farms—an important pathway toward inclusive aquaculture health services. Most research to date addresses human and veterinary applications; end-to-end systems for specific aquatic pathogens remain underdeveloped.

#### 3.3.3. Regional Epidemic Mapping and Big-Data Early Warning

Once a cloud platform aggregates multi-site testing data, it can generate spatiotemporal heat maps of pathogen positivity rates, help characterize the direction and apparent rate of disease spread, and provide data support for regional joint prevention and control, seed movement regulation, and culling decisions—achieving a functional leap from “individual diagnosis” to “epidemiological surveillance.” The European Union has already established aquatic animal health surveillance networks covering its member states (such as the Animal Disease Information System, ADIS), and WOAH promotes global sharing of aquatic animal disease surveillance information through WAHIS (World Animal Health Information System); the networked deployment of POCT would supply these systems with high-volume frontline data and help address gaps in grassroots surveillance.

#### 3.3.4. Closed-Loop Linkage with Farm Management Systems

Overlaying POCT data on the same platform with water quality (dissolved oxygen, pH, ammonia nitrogen, and temperature), feeding, and meteorological data enables predictive models of “environmental stress—pathogen load—disease risk”, moving disease prevention from “reactive” to “predictive.” Machine-learning early-warning models for farmed-fish disease have been demonstrated in marine aquaculture (e.g., a cryptocaryoniasis early-warning system; Xie et al. [87]), and the data infrastructure for health management in Norwegian, Scottish and Irish salmon production is maturing [84], but the specific fusion of pathogen POCT data with environmental and production streams into routine risk prediction remains at an early pilot stage, representing the higher-order form of precision fisheries and smart biosecurity.

Overall, the convergence of POCT with smart fisheries remains at a stage of scattered demonstrations, with Europe, North America, and China among the more active adopters, and a clear gap remaining before a routine surveillance network covers the major farming regions.

### 3.4. Representative Application Cases Worldwide

Table 4 presents a synthesis framework for POCT applications across the world’s production regions and operational scenarios. It should be noted that the citation strength of the individual cases in the table varies: some derive only from public methodological literature or company-disclosed information, and some still require concrete scaled-operation data; their sources should therefore be interpreted with caution.

Overall, the application of POCT in global fish disease control has moved from “laboratory technology validation” to “localized small-scale field deployment”, producing practice models adapted to varying levels of industrial development across pond-side screening, border control, and digitalized biosecurity. Yet the gaps between “usable” and “easy to use” and between “demonstration” and “universal adoption” remain wide, and global practice still faces multiple barriers—sample matrix compatibility, environmental stability, standardization systems, and cost and accessibility—which are precisely what the next section will systematically analyze.

## 4. Principal Challenges and Bottlenecks Facing Global Scale-Up

Although POCT has demonstrated practical value in global aquaculture, moving from “laboratory-verified feasibility” to “industry-scale usability”, and from isolated pilots to equitable worldwide deployment, still requires confronting a composite set of bottlenecks spanning sample matrices, technical performance, field environments, industrial structure and institutions. These challenges are global but manifest differently across regions and income levels. The fifth category addresses two widely overlooked shortfalls—regulatory standing and interdisciplinary talent—that fundamentally shape who can access reliable diagnosis and on what terms.

### 4.1. Matrix Interference Across Production Systems

Under laboratory conditions, samples undergo strict pre-processing—centrifugation, spin-column purification, dilution, and quantification—holding matrix interference to a low level. At culture sites worldwide, however, the obtainable samples are far more complex, a foundational technical challenge across production regions. A recent review of aquaculture immunoassays [88] identifies “matrix interference, field robustness and real-world validation” as the core engineering challenge in moving this field from laboratory to farm [88].

Across the world’s different farming systems, the sources and severity of matrix interference differ substantially:

1. Freshwater pond-culture regions (major carp cultures of China, South and Southeast Asia): culture water is rich in suspended particulates, algae, humic substances and chemical residues. Humic acids directly inhibit polymerase-mediated amplification and quench dye fluorescence [89]; multi-component inhibition of qPCR by humic/fulvic acids, polysaccharides, metal ions and nucleases in environmental water concentrates is systematically documented [89]; and sediment heavy metals and humic matter may further complicate detection [89,90].
2. Marine and brackish cage-culture regions: represented by salmon farming in Norway, Chile and Scotland and by marine finfish culture in the Mediterranean and Southeast Asia, where high ionic strength, biofouling and variable dissolved organic matter can alter biorecognition and signal stability, so performance should be validated across realistic salinity and matrix gradients rather than extrapolated from buffer tests [88]. Extracellular polysaccharides, dissolved organic matter and planktonic microbial communities in seawater may additionally contribute to non-specific adsorption and amplification inhibition [91].
3. Industrialized recirculating aquaculture systems (RASs). Although water quality is controllable, sloughed biofilm material, microbial metabolites and disinfection by-products still interfere with trace-level detection; and in high-density culture, the body mucus and gill-filament tissues of fish are rich in mucopolysaccharides and contain proteases that may compromise antibodies or enzymes in test reagents—a detection difficulty common to industrialized aquaculture worldwide.
4. Moribund and decomposed samples: in tropical regions, endogenous nucleases remain highly active in moribund fish at high ambient temperature, so pathogen nucleic acid integrity degrades rapidly; combined with absent cold-chain protection during field sampling, this may contribute substantially to false negatives. This inference is mechanistically plausible (nuclease activity correlates with temperature), but, to our knowledge, no dedicated survey has quantified the hypothesized severity gradient between low- and high-latitude regions.

Matrix characteristics vary enormously across species and farming modes, and no universal pre-processing protocol exists. The result is significant variability between ponds, operators and regions—a variability that ultimately disadvantages farmers who depend on a single, low-cost test for a consequential management decision.

### 4.2. Field-Environment Adaptability and Regional Disparities in Reagent Storage

Aquaculture sites are the most demanding “operating theatre” for POCT devices and reagents. Temperature, humidity, electricity and cold-chain conditions differ enormously between the world’s climate zones and between regions of different economic standing, so that environmental robustness is inseparable from questions of access: the technical burden of harsh conditions falls hardest on the regions least able to afford compensating infrastructure, producing a differentiated set of challenges.

1. High temperature and humidity: pond-side temperatures in tropical and subtropical zones routinely reach 35–40 °C. Elevated temperature accelerates enzyme inactivation, antibody denaturation and fluorophore quenching; humidity can alter strip capillary flow, potentially causing uneven development or false-positive lines; dust and sea spray contaminate optical and electrical interfaces. Quantitative evidence remains sparse: unprotected reagents can lose activity within days at 25–37 °C, whereas protectant-optimized lyophilized formulations can retain performance for weeks at ambient temperature [62]; product-level accelerated-stability data nevertheless remain scarce [61,62].
2. Low temperature and offshore operations: in the temperate and cold-water marine culture regions of Norway, Canada and Chile, winter freezing poses a risk of reagent damage; offshore cage platforms are cramped, subjected to severe salt-spray corrosion and constrained in power supply, which makes stable operation of precision instruments and temperature-controlled reagent storage difficult.
3. Cold-chain and logistics breaks: the “last hundred kilometers” between manufacturer and pond is an under-recognized risk to reagent performance. It has been argued that “disparities in cold-chain accessibility exacerbate global inequity in infectious disease control”: incomplete cold chains, poor distributor storage and refrigeration-free last-mile delivery can occur in resource-limited regions, while even wealthy regions face long logistics cycles to remote fishing grounds and island farms. Products that leave the factory calibrated may deviate before reaching the users least able to buffer against it [60].

The industry’s response—lyophilized reagents, room-temperature-stable enzymes, and thermostable antibodies—is advancing, but published room-temperature shelf lives remain on the order of weeks to months (lyophilized PCR reagents stable 1.5 months at 25 °C and 6 months at 4 °C; optimized formulations 14–30 days at 37 °C; studies cited by Talbot [60]), and few aquaculture POCT products have published evidence of full cold-chain independence. Independent verification against such reports is therefore advisable.

### 4.3. Absence of a Global Standardization and Regulation Framework

The global fish-disease POCT market faces “many products, few yardsticks, and highly uneven quality”. The absence of standardization is an institutional bottleneck that constrains healthy industry development, and it presents a stratified pattern across economies of different development levels.

1. International level: the WOAH Manual of Diagnostic Tests for Aquatic Animals [18] prescribes validated laboratory methods—cell culture, real-time PCR, sequencing, and in situ hybridization—for listed diseases. LAMP is described for WSSV (Chapter 2.2.8) but is not incorporated into the KHVD chapter; a confirmed case requires clinical observations plus virus isolation or PCR/sequencing, so a positive LAMP result alone cannot confirm a case. The Manual states that commercial kits are acceptable only if validated as fit for their intended purpose, and WOAH’s diagnostic-kit registration activities are suspended from May 2024 until May 2027—leaving, for a defined window, no active WOAH international register for on-site aquatic diagnostics [53].
2. Advanced economies: the European Union, the United States and Japan have comparatively well-developed regulatory systems for veterinary medicines and biologics, but dedicated approval pathways for aquaculture POCT are often unclear, and products may fall under general veterinary diagnostic or in vitro testing frameworks, with long and costly market-access cycles that will suit a sector characterized by many target species and rapidly changing products.
3. Emerging and developing economies: market-entry pathways for aquaculture POCT are heterogeneous across China, Southeast Asia and Latin America, and some products circulate as “rapid test reagents” without transparent registration requirements. Quality-assessment infrastructure remains laboratory-centered: inter-laboratory ring trials exist for reference methods (e.g., IPNV detection in Chile; Tapia et al. [92]), and Bayesian latent-class field evaluations have been conducted for salmonid alphavirus in Norway [30], but we found no reports of IQC/EQA programs dedicated to on-site rapid aquaculture diagnostics.

The direct consequence of this standards vacuum is a global risk that weakly validated, lower-cost products may displace better-validated alternatives on price, thereby eroding end-user trust—a deep institutional constraint on scale-up. Where no transparent performance yardstick exists, resource-poor buyers are least protected from substandard reagents; a review of nanomaterial-enhanced lateral-flow assays for aquaculture similarly identifies scalable synthesis cost, batch stability and shelf life as the binding constraints [72].

### 4.4. Cost Pressure at the Grassroots Level and the Awareness Divide

Whether technology takes root at the front line depends on affordability, operability, trust and durable uptake, and the principal contradiction differs by income level. This synthesis is based on industry-logic inference rather than direct measurement: adoption studies among smallholder fish farmers in East Africa [59] and Kenyan health-management surveys [56] document general technology-adoption constraints, but no published survey quantifies willingness to pay specifically for aquatic-animal diagnostics [56,59].

1. Balancing cost and benefit: in smallholder-dominated regions of Southeast Asia, South Asia and Africa, thin margins may cap willingness to pay for a single test—an affordability gap may exclude farmers from molecular POCT even when test performance is adequate. In middle-income regions such as China and Latin America, the ratio of per-test cost to farming income can be prohibitive: unless testing frequency rises, the early-warning value of diagnosis cannot be realized, risking a negative feedback loop of “cannot afford → do not use → technology does not improve”. In Norway and Chile, labor costs may outweigh consumable costs, so testing must be streamlined and largely unattended.
2. Equipment investment and access: portable fluorometers and isothermal instruments exceed the means of individual households. In developing countries, access is mediated through cooperatives, extension programs and enterprise-procured shared equipment, leaving coverage thin; in developed countries, corporate farms own instruments but remote small holdings still fall outside coverage. Equipment access is itself an equity variable—diagnostic capability is determined as much by institutional arrangements for sharing capital equipment as by the technology itself.
3. Operating knowledge and training systems: even “foolproof” strips demand rigor in sampling site, tissue quantity, dilution and timing, which directly determine accuracy. Front-line training still relies mostly on ad hoc manufacturer instruction; systematic technology-extension systems may be limited. The cognitive threshold is deeper still: farmers habituated to empirical treatment will convert to etiological diagnosis only after it delivers a tangible, perceptible reduction in losses.

This means that the global promotion of POCT may not rely on product sales alone; it may need to be bundled with integrated health-management service packages spanning “diagnosis → prescription → medication → follow-up” if sustained frontline adoption is to be achieved—an insight that is as much about equitable service delivery as about business-model design.

### 4.5. The Dual Global Shortfall in Regulatory Standing and Interdisciplinary Talent

Beyond the four technical and industrial bottlenecks above, there are two further, commonly overlooked shortcomings affecting economies worldwide.

Regulatory level: in most countries, statutory methods for notifiable diseases remain laboratory-based—virus isolation, qPCR and ELISA—and WOAH standards require confirmation through prescribed pathways. The legal standing of POCT results in administrative regulation, border quarantine, trade arbitration, insurance and seed-transaction disputes therefore remains undefined, weakening their value for independent use.

- The European Union and the United States have relatively mature regulatory systems, yet official use of POCT in quarantine still requires separate validation and authorization;
- Developing countries are generally in a regulatory vacuum, with neither entry requirements nor rules for the acceptance of results.

Establishing tiered, category-based, and emergency-prioritized mechanisms for the official acceptance of POCT results is a common global challenge for unlocking the institutional benefits of the technology.

Talent level: a globally insufficient supply of interdisciplinary expertise. The research, development and service provision of fish-disease POCT sit at a highly interdisciplinary junction: they require familiarity with fish pathology and epidemiology, command of molecular biology, microfluidics, materials science, signal detection and AI algorithms, and the ability to engage directly with the real needs of the production front line.

- Developed countries may have greater research capacity, but extension and advisory systems—documented as a structural weak point of aquaculture development in both high-income and low-income settings [93]—face staffing, funding, and knowledge-renewal constraints that limit the “last mile” of technology promotion and maintenance [57];
- Developing countries suffer a dual scarcity of both research talent and front-line extension personnel, and the fragmented disciplinary structures of their universities leave the supply of composite, cross-boundary talent severely inadequate.

This structural contradiction in talent supply is a deep-seated factor constraining both the technological iteration and the industrial deployment of POCT worldwide, and it is also a key reason why the diagnostic-capability gap between regions persists despite the availability of suitable technologies.

### 4.6. Global Summary

The five bottleneck categories discussed above are summarized by shared constraints, regional patterns and priority responses in Table 5.

It can be seen that these challenges are interwoven and mutually amplifying: without standardization, products cannot win large-scale procurement; without large-scale procurement, costs cannot be spread; without affordable costs, grassroots adoption cannot advance; and without the data generated by grassroots adoption, products and standards cannot be refined in return. Breaking this closed loop requires global, coordinated efforts in technology development, standard-setting, policy guidance, industrial organization and talent cultivation.

## 5. Future Trends and Prospects from a Global Perspective

### 5.1. Multiplexed Co-Detection of Targets (Multiplexing): From “One Disease, One Test” to a “Multipathogen Profile”

The frequent occurrence of mixed infections is a central limitation of single-target POCT: bacterial septicemia may coexist with viral, parasitic and bacterial infections, as well as environmental stressors. Next-generation POCT therefore aims at “many assays on one strip, many targets in one tube”—screening multiple pathogens or biomarkers in a single sampling and reaction.

Technologically, multiplexing is advancing along the following three parallel routes:

- Arrayed lateral-flow immunoassay. Multiple test lines on one strip, or fluorescent-microsphere encoding, enable multi-target detection; validated precedents exist in food safety, where a 10-channel up-converting phosphor lateral-flow disk detected ten foodborne pathogens within 20 min without cross-reactivity [76]. For aquaculture pathogens, however, existing LFIA products remain overwhelmingly single-target: a review of 22 devices shows multi-pathogen arrayed products are still rare [75]; the closest verified steps are a dual-genotype (RGNNV/SJNNV) nanobiosensor [32] and whole-virion nanoparticle strips [33]—region-customized combination strips are a reasonable expectation, not an existing reality.
- Multiplexed isothermal amplification with multi-channel readout. Multiple primer sets are introduced into LAMP/RPA systems to achieve “many targets in one tube,” combined with microfluidic multi-channel or fluorescence-encoded readout. Multiplex LAMP/RPA has accumulated extensive methodology in human medicine and food safety, and migration to aquaculture pathogen panels is under way, but inter-primer interference and the balancing of sensitivity across targets remain recognized engineering hurdles.
- Multiplexed CRISPR diagnostics. RPA/RAA–Cas12a systems have been reported for a growing number of aquaculture pathogens—including WSSV, AHPND, EHP, scale drop disease virus (SDDV), infectious spleen and kidney necrosis virus (ISKNV), mandarin fish ranavirus (MRV), cyprinid herpesvirus 2 (CyHV-2), spring viraemia of carp virus (SVCV) and tilapia lake virus (TiLV)—with reported limits of detection of 1–200 copies and end-to-end run times of 40 min–2 h, including one-pot and lyophilized single-tube formats [7,16,17,58]. The demonstrated aquaculture multiplex level is currently two-target: a simultaneous visual WSSV/EHP assay [6] and a duplex dRAA–Cas12a assay for the aerA/hlyA genes of *A. hydrophila* (2 copies, ~45 min) [24]. Three-to-five-pathogen panels remain confined to human medicine; no scaled commercial aquaculture product has yet landed.

The shared challenge of multiplexing is signal crosstalk, cross-reactivity and sensitivity equilibration across targets, requiring optimization at primer/antibody design, reporter chemistry and surface chemistry. As engineering and validation mature, dual-to-few-target products tailored to regional pathogen panels (e.g., “grass carp GCRV + *A. hydrophila*” or “shrimp WSSV + AHPND + EHP”) may take shape—a reasonable inference whose timing depends on engineering breakthroughs.

### 5.2. Digitization, Miniaturization, and AI-Assisted Diagnosis: From Visual Test Strips to Lab-on-a-Chip Decision Support

The core vehicle of miniaturization is the microfluidic lab-on-a-chip: sample pre-treatment, lyophilized reagent storage, reaction and detection integrated on a centimeter-scale chip with disposable cartridges and a compact reader, achieving “sample-in—result-out” automation. Commercialization still consists of a handful of device-level products alongside laboratory prototypes (Section 2.2.4); mass production and widespread adoption remain mid-term expectations over the 3–5-year horizon.

Intelligentization is proceeding simultaneously at the device level and the cloud level:

1. On-device intelligence: ARM-/MCU-based systems on chip perform thermal control, fluidic actuation, optical/electrochemical signal acquisition, and preliminary interpretation, allowing devices to operate independently without reliance on the cloud—a fit for remote producing areas with weak power infrastructure and for offshore operations. This is a common upgrade direction for portable devices worldwide.
2. Cloud + AI intelligence: test results are fused with temporal, spatial, water-quality, meteorological and epidemiological data to predict outbreak risk and optimal intervention timing. Digital linkage in global smart fisheries currently operates on environmental and production data—Mowi 4.0 and the Google-X-origin Tidal platform use AI-powered underwater cameras across 250+ net-pens for real-time biomass, feeding and sea-lice monitoring [94]—whereas routine linkage of POCT pathogen data with farm-management systems is not yet publicly reported: the salmonid health-management literature [84] and the Barentswatch Fish Health platform still rely on laboratory and observer-reported diagnoses. Such linkage is an emerging trend, not an achieved reality.

Generative AI and multimodal models are giving rise to “aquaculture veterinary assistants”: a farmer photographs a diseased fish and uploads the strip result, and the model outputs a probable pathogen spectrum with management recommendations. The evidence base is early: CNN image diagnosis reports 88–97% classification accuracy in laboratory studies [83], but a recent narrative review concludes such models have not been systematically validated across farm sites, imaging conditions and disease stages and should be positioned as decision support rather than veterinary replacement [95]. Reliability validation, hallucination risk and liability boundaries remain unresolved before deployment.

### 5.3. Cross-Disciplinary Innovation in Novel Materials and Chemical Biology: The Material Basis for Cold-Chain Independence and Enhanced Sensitivity

Progress at the performance frontier of POCT increasingly relies on advances in materials science. To address the three shared bottlenecks of cold-chain independence, matrix-interference resistance and sensitivity enhancement, cross-disciplinary innovation in novel materials and chemical biology is advancing on multiple fronts.

1. Engineered enzymes. Directed evolution or AI-assisted design of Bst polymerase and Cas proteins can yield ultra-stable variants tolerant of heat, salt and inhibitors. In aquaculture, RPA-CRISPR enzymes and primers lyophilized into a single tube for MRV/ISKNV detection demonstrate feasibility of the “lyophilization + on-site reconstitution” route, but systematic engineered-enzyme studies for aquaculture remain early: stabilization know-how sits mostly in proprietary formulations, patents and adjacent-domain open-source work, not in peer-reviewed aquatic studies [17,62].
2. Nanozymes and nanocatalysts. MOF-based nanozymes and related composites have enabled sensitive colorimetric detection of foodborne bacteria and small-molecule contaminants in adjacent food-safety applications and can be integrated with portable or smartphone-assisted readouts [71,96,97]. To date, evidence for nanozyme-based pathogen detection is concentrated in food safety and clinical diagnostics, and reports of direct application to aquaculture pathogens remain scarce—their migration to aquaculture diagnostics is a forward-looking judgment grounded in a solid adjacent-domain basis.
3. Synthetic biology components. Toehold-switch RNA sensors combined with lyophilized cell-free systems enable an “add-sample—read-color” mode of nucleic acid detection on paper substrates: the route was validated for Zika virus [98] and achieved 98.5% diagnostic accuracy comparable to RT-qPCR in field trials across five Latin American countries [99], with lyophilized components stable at room temperature for more than one year. This paradigm provides a technological template for future instrument-free, self-reporting field tests in aquaculture; however, toehold-switch sensors for aquatic pathogens have not yet been reported in the peer-reviewed literature—development activity remains concentrated on human and plant viruses (reviewed by Geraldi et al. [100])—marking a clearly defined, and defensible, research gap.
4. Novel reporter systems. Aggregation-induced emission (AIE) molecules, up-converting phosphor nanoparticles (UCNPs), and time-resolved fluorescence can markedly improve signal-to-noise ratios and photobleaching resistance—UCNPs have been validated as the core reporter label in the 10-channel lateral flow assay for foodborne pathogens [76], so migration to aquaculture diagnostics is underpinned by the materials themselves.
5. Wearable and flexible sensing. These systems can complement POCT by continuously monitoring host physiology and environmental exposure rather than directly identifying pathogens. Kaidarova et al. [101] reviewed this emerging field, and the implantable “Lab-on-a-Fish” tag demonstrated real-time telemetry of fish heartbeat, tail-beat, position and environmental parameters [102].

Overall, the industrial translation of these frontier results could support POCT advances at the three long-standing constraint points of cold-chain independence, sensitivity limits, and matrix interference resistance.

### 5.4. Deep Integration into a System of Precision Antimicrobial Use and Green Disease Control

The ultimate value of POCT lies not merely in “detecting the pathogen” but in driving global aquaculture from experience-based diagnosis and treatment towards precision decision-making—a transition that sits at the heart of the One Health agenda, of antimicrobial stewardship, and of the sustainable intensification of global food production. The policy background is firmly documented in the international literature: in 2015, the sixty-eighth World Health Assembly endorsed the Global Action Plan on Antimicrobial Resistance (GAP), developed jointly with FAO and WOAH [103]; FAO subsequently issued its FAO Action Plan on Antimicrobial Resistance (2016–2020), which explicitly incorporated the aquaculture sector and carried this commitment forward in the 2021–2025 action plan [104]; and the United Nations General Assembly high-level meeting on antimicrobial resistance of September 2024 adopted a political declaration committing to a substantial reduction in antimicrobial use in agriculture and food systems globally by 2030 [105].

1. Integrated pathogen diagnosis + rapid antimicrobial susceptibility testing. A systematic analysis of AMR national action plans in 95 countries found that 37% omit aquaculture entirely [39]—a governance gap that precision diagnosis can help close, although it can support surveillance and stewardship where aquaculture is included in those plans. Isothermal-amplification- and CRISPR-based resistance-gene detection and microfluidic rapid susceptibility testing have well-established methodological foundations in human and veterinary medicine, including CRISPR platforms for antibiotic-resistance-gene analysis [106], yet aquaculture-specific point-of-care ARG devices remain unvalidated in production settings—consistent with the sector-wide AMR surveillance deficit [39,40].
2. Treatment monitoring and withdrawal-period determination. Food-safety regimes impose stringent requirements on drug residues, and major economies including the European Union, the United States, and China have all established maximum residue limit (MRL) standards for aquatic products. POCT enables periodic pathogen monitoring during treatment, typically qualitative or semi-quantitative, to inform veterinary reassessment and guide corresponding rapid interventions. Withdrawal periods, however, must remain governed by authorized product labels and residue regulations; rapid residue tests before harvest may support screening for maximum-residue-limit compliance but do not replace confirmatory analytical methods.
3. Synergy with green control measures. Regional portfolios differ: vaccination and probiotics are prominent in several high-income systems, while immunostimulants, phytogenic products and biological-control approaches are being evaluated or applied across Asia–Pacific and Latin American aquaculture [41]. POCT can form a “diagnosis—decision—intervention” closed loop with the full range of green measures: for instance, identifying a viral disease by POCT and then supporting strict isolation while avoiding inappropriate antibiotic use, thereby supporting global antimicrobial-reduction targets and sustainable intensification.
4. Alignment with the global Sustainable Development Goals. As an enabling technology for precision disease control, POCT can contribute to the UN SDG framework: food security (SDG 2), health—read here through a One Health lens (SDG 3), responsible production and consumption (SDG 12), clean water and sanitation (SDG 6), and life below water (SDG 14).

### 5.5. An Indicative 5–10 Year Outlook for POCT in Global Aquatic Animal Disease Control

Integrating the technological and industrial trends above permits a staged outlook for the development of fish disease POCT worldwide over the next 5–10 years. It must be emphasized that Table 6 presents indicative scenarios reasoned from current R&D progress and the regularities of industrialization—they are not verifiable predictions; the attainment of each time window depends on the pace at which the bottlenecks described in Section 4 are resolved.

## 6. Conclusions: Positioning POCT as Sustainable-Transition Infrastructure for Global Aquatic Animal Health

The preceding sections support a conclusion that is uncomfortable for a technology-centered reading but essential for a sustainability-centered one: the binding constraints on POCT for global fish health are not only field analytical reliability but also deployment, governance and equity. The platforms reviewed in Section 2 already span the sensitivity–cost–simplicity space from subsistence ponds to industrial sea-cages. What blocks their sustainability dividend is a chain of socio-technical failures: matrix interference that erodes field reliability, cold-chain dependence that excludes tropical and remote regions, absent performance standards (compounded by the WOAH diagnostic-kit register suspension [53]), regulatory systems that grant POCT results no legal standing, and knowledge gaps at smallholder and extension levels. Diagnostic capability currently follows economic geography rather than disease burden—the inverse of what One Health and the SDG demand [53].

Viewed through the sustainability lens adopted here, POCT is best understood not as a laboratory instrument shrunk to pocket size but as enabling infrastructure for the following three interlocking transitions in blue food production: environmental—antimicrobial stewardship at the point of need, given that 37% of national AMR action plans omit aquaculture entirely and that the 2024 UN political declaration commits to measurable reductions in agricultural antimicrobial use by 2030; economic—loss avoidance and trade resilience, for which the decisive question in middle-income producing regions is cost-structure engineering rather than the next order-of-magnitude gain in limit of detection; and social—equitable epizootic intelligence, in which connected readers and geotagged, cloud-aggregated results can feed WOAH WAHIS and national early-warning systems, subject to data sovereignty, accuracy governance for AI-assisted interpretation, and One Health safeguards against the circulation of antimicrobials and resistance determinants.

Realizing this positioning requires reorienting how success is measured. For researchers, the priority is field-validation-first design—matrix robustness, tropical shelf life and operator error by non-specialists, benchmarked against reference methods on unselected production samples. For standard setters, the 2024–2027 register-suspension window is the opportunity to build a fit-for-purpose performance-evaluation standard with graded claims (screening, presumptive and confirmatory), reference panels and proficiency testing. For policymakers, the tasks are to integrate POCT into national AMR action plans and to define the legal weight of point-of-care results by claim grade. For industry, competition should shift to total deployment value—cold-chain-independent reagents, robust instrument-free readout and consumable pricing compatible with smallholder economics. The staged outlook in Table 6 illustrates how these enabling technologies could advance towards equitable aquatic disease surveillance under appropriate governance.

## 7. Limitations of This Review

### 7.1. Limitations of the Available Evidence

Most reported performance metrics derive from developmental studies or single-site validation rather than prospective testing of unselected production samples. Field conditions can reduce diagnostic performance, particularly when crude samples contain amplification inhibitors. Detection limits are also reported using inconsistent units and templates, while confidence intervals are often absent, preventing direct comparison across studies. Moreover, reference methods are not always definitive, and single-laboratory validation leaves inter-operator variability largely unassessed. The lack of reported real-world costs further limits evaluation of affordability.

### 7.2. Limitations of the Review Methodology

This is a narrative rather than a systematic review. Although the search strategy, inclusion criteria and evidence grading are documented in Section 1.4, the protocol was not prospectively registered, no formal risk-of-bias instrument was applied and no meta-analysis was performed. Heterogeneity in study designs and reported outcomes meant that quantitative findings reflect reported ranges rather than pooled estimates. The reliance on the English- and Chinese-language literature, together with limited peer-reviewed evidence for some industry and regulatory claims, may have introduced selection bias. Publication bias towards successful assays and the geographic concentration of validation studies also limit the generalizability of current evidence, particularly in African and Latin American settings.

## Figures and Tables

**Table 1:** Major infectious diseases of farmed aquatic animals and the POCT formats reported for their early detection.

Disease (Pathogen)	Principal Hosts	WOAH-Listed	Specimen Used on Site	POCT Format Reported	Reaction Time	Ref.
Crustaceans						
White spot syndrome (WSSV)	*Penaeus vannamei*, *P. monodon*	Yes	Pleopod, gill, hemolymph, post-larvae	Insulated isothermal PCR (POCKIT); RPA—LFD; multiplex CRISPR-Cas	20–60 min	[4,5,6]
Acute hepatopancreatic necrosis disease (*V. parahaemolyticus*, pirA/pirB+)	*P. vannamei*, *P. monodon*	Yes	Hepatopancreas, stomach, pond water	RPA—CRISPR/Cas12a—LFD; lateral-flow immunoassay (LFIA)	≤2 h; ~20 min	[7,8]
Hepatopancreatic microsporidiosis (*Enterocytozoon hepatopenaei*)	*Penaeid shrimp*	No	Hepatopancreas, feces, pond water/soil	RPA; RPA—LFD; multiplex CRISPR-Cas	20–45 min	[6,9,10]
Freshwater finfish						
Grass carp hemorrhagic disease (GCRV)	Grass carp, black carp	No	Kidney, spleen	Colloidal-gold GICA strip; RT-LAMP—LFD	10–60 min	[11,12]
Koi herpesvirus disease (KHV; CyHV-3)	Koi, common carp	Yes	Gill, kidney, mucus	LAMP; PCR—LFD; fluorescence real-time LAMP (pond-side test)	30–60 min	[13,14,15]
Spring viraemia of carp (SVCV)	Cyprinids, ornamental fish	Yes	—	RAA—CRISPR/Cas12a	~45 min	[16]
Megalocytivirus infection (ISKNV/RSIV/TRBIV)	Mandarin fish, sea bream, grouper	Yes (Megalocytivirus pagrus1)	Spleen, kidney	RPA—CRISPR/Cas12a; LAMP	~60 min	[17,18]
Tilapia lake virus disease (TiLV)	Nile tilapia and hybrids	Yes	Liver, brain, gill	RT-RPA—Cas12a; RT-RPA–LFD	<1 h; ~20 min	[19,20]
Motile aeromonad septicemia (*Aeromonas hydrophila*)	All freshwater finfish	No	Kidney, blood, water	Microfluidic LAMP; electrochemical aptasensing; RAA—CRISPR/Cas12a	~45 min	[21,22,23,24]
Streptococcosis (*S. agalactiae*, *S. iniae*)	Tilapia, sea bream, catfish	No	Kidney, brain, blood	FTA-card colorimetric LAMP; LAMP	30–60 min	[25,26]
Edwardsiellosis (*Edwardsiella* spp.)	Catfish, eel, tilapia	No	Kidney, spleen	Electrochemical aptasensing; surface-enhanced Raman scattering (SERS)	~45 min; 20 min	[23,27]
Marine finfish						
Infectious salmon anemia (ISAV, HPR-deleted/HPR0)	Atlantic salmon	Yes	Kidney, heart, gill	On-site RT-PCR/iiPCR; no validated LAMP or RPA identified	—	[28]
Viral hemorrhagic septicemia (VHSV)	Salmonids, marine finfish	Yes	Kidney, heart	RT-LAMP	~60 min	[29]
Pancreas disease (salmonid alphavirus, SAV)	Atlantic salmon, rainbow trout	Yes	Heart, kidney	Field-evaluated RT-qPCR	—	[30]
Viral nervous necrosis (betanodavirus; ICTV)	Sea bass, grouper, flatfish, barramundi	No	Brain, eye, larvae	Amplification-free microfluidics; lateral-flow strip biosensor; nanoparticle LFIA	30 min	[31,32,33]
Vibriosis (*V. parahaemolyticus*, *V. harveyi*, *V. anguillarum*)	Marine finfish, shrimp	No	Kidney, water, seafood	Surface-enhanced Raman scattering (SERS); SERS nanoprobes	20 min; 30–60 min	[27,34,35]

Note: WOAH-listed status follows the WOAH Aquatic Animal Health Code (2026) [18]. “—” indicates that no value was reported in the cited source.

**Table 2:** Comparison of conventional laboratory diagnosis with the operational requirements of on-site, frontline aquaculture testing.

Dimension	Traditional Laboratory Diagnosis	Practical Needs of On-Site/Frontline Aquaculture *	Representative References
Turnaround time	Pathogen culture takes 3–7 days; qPCR/ELISA requires several hours to 1 day	“Immediate testing upon disease onset, results available on the same day”, preferably within 30 min	Conventional: [14,30,18,48]; on-site target: [5,49,50,51,52]
Equipment	Requires PCR instruments, microplate readers, centrifuges, ultra-low temperature freezers, etc.	Instrument-free or battery-powered formats are preferred	Conventional: [30,18,53]; on-site target: [4,50,51,52,54,55]
Personnel	Requires technicians with professional training in molecular biology or microbiology	Usable by farmers and technicians after brief training	Conventional: [53,56,57]; on-site target: [51,52,55,58,59]
Site	Requires a standard PCR laboratory (with zoned areas and clean conditions)	Suitable for use at pond sides, in farm workshops, or aboard vessels	Conventional: [18,53]; on-site target: [4,5,14,51,52]
Sample handling	Requires cold-chain transport (4 °C/−20 °C) under strict storage conditions	Room-temperature transport, where validated, with direct or minimally processed on-site sampling	Conventional: [30,60]; on-site target: [25,52,61,62]
Cost per test	Relatively high (including equipment depreciation, reagents and labor)	Preferably several US dollars or less per test	Conventional: [58,63]; on-site target: [51,52,59]

Note: * The entries in this entire column are operational targets compiled from published field evaluations and from the WHO ASSURED/REASSURED criteria for decentralized testing [51,52]; they are indicative design goals and not validated performance specifications.

**Table 4:** Representative applications of POCT across aquaculture regions and operational scenarios.

Application Scenario	Core Production Region	Technology Combination	Application Form	Public Evidence Strength	Representative Referenced
Early-onset self-testing in major freshwater fish regions	Yangtze/Pearl River basins of China; Southeast Asian freshwater farming regions	GICA/FIA strips; portable LAMP/RPA devices	Pond-side results in 15–60 min to inform isolation, feed cessation, and targeted treatment decisions	Strong methodological evidence; limited evidence from scaled operations	[11,12,13]
Routine monitoring and disease screening in shrimp farming	Thailand, Vietnam, Ecuador, Indonesia	WSSV/AHPND colloidal gold strips + RPA-CRISPR/Cas12a + lateral-flow strips	Pond-side/workshop rapid testing, seed batch sampling, intensified monitoring during outbreaks	Methodological evidence reported; independent adoption-rate data remain limited	[4,5,6,7,8,55,77,78]
Pathogen monitoring in marine salmon cages	Norway, Chile, Scotland, Canada	For ISAV, IHNV, SAV/PDV, IPNV, and related pathogens, RT-PCR/qPCR/ddPCR are dominant; iPCR/LAMP are exploratory, and RPA remains early-stage	Regular health monitoring at sea-based sites, partly paired with laboratory confirmation	Strong laboratory-testing evidence; limited evidence on scaled portable POCT application	[28,30,87]
Disease control in international ornamental fish trade	European Union, Japan, China, Southeast Asia	CyHV-3/SVCV colloidal gold rapid tests + port laboratory qPCR confirmation	Two-tier “rapid screening—confirmatory backstop” quarantine workflow	Industry practice has been reported; systematic regulatory evidence remains limited	[14,15,16]
Emerging smart-fishery disease-surveillance pilots	China, Norway	IoT smart strip readers + cloud GIS management platform	Automated upload of test data to support generation of regional epidemic heat maps	Pilot projects exist; limited evidence of systematic deployment	[54,83,87]
Batch quarantine for shrimp exports	Thailand, Vietnam, Indonesia, Ecuador	LAMP/RPA isothermal amplification + lateral-flow strips	Pre-export factory self-testing, port spot verification	Industry-reported practice; independent or systematic evidence remains limited	[9,10,82]
Biosecurity control at national aquatic broodstock and breeding farms	Major aquaculture breeding countries worldwide	Lyophilized RPA + lateral-flow strips, ambient-temperature reagent transport	Three checkpoints: testing at entry, during spawning/rearing, and at exit	Relatively favorable infrastructure at large-scale breeding facilities; limited evidence of uptake at the smallholder level	[4,55]

Note: The “public evidence strength” column indicates the citable evidence underpinning each case row—“strong” means the peer-reviewed literature or authoritative institutional public data exist, while “limited” means only scattered reports, company disclosures, or industry convention exist.

**Table 5:** Global bottlenecks to aquaculture POCT scale-up and priority responses.

Bottleneck Category	Core Contradiction Shared Globally	Pattern of Regional Differentiation	Most Urgent Direction for Breakthrough	Representative References
Complex sample matrices	Field samples are “dirty” and heterogeneous, causing false negatives/positives and result variability.	Humic-substance interference may be particularly prominent in tropical freshwater regions, whereas high-salinity interference may predominate in marine regions.	Matrix-tailored sample pre-processing + inhibitor-resistant enzyme systems + high-specificity recognition elements.	[88,89,90]
Field-environment adaptability	Uncontrolled temperature, humidity, power supply, and cold-chain conditions can destabilize reagent and instrument performance.	Cold-chain disruptions may be more prevalent in developing countries, whereas offshore operational constraints may be more pronounced in developed countries.	Lyophilized reagent technology + room-temperature-stable biological reagents + low-power integrated devices.	[47,61,62]
Absence of standardization	No unified yardstick for product performance; the market is uneven and risks “bad money driving out the good”.	Regulatory requirements may be rigorous but slow to implement in developed countries, whereas standards may be lacking in some developing countries.	Internationally recognized standards + independent third-party evaluation + a global external quality assessment (EQA) scheme.	[18,53]
Grassroots cost and awareness	Costs, training, and service provision are insufficiently aligned, making deployment difficult and limiting uptake.	Low-income countries may be more sensitive to direct costs, whereas developed countries may be more sensitive to labor costs.	Service-based business models + shared testing systems + grassroots capacity building.	[56,57,59,93]
Regulation and talent	The legal status of diagnostic results is often undefined, and the supply of interdisciplinary talent is insufficient.	Regulatory regimes differ widely across countries; talent is unevenly distributed globally.	Clear, tiered mechanisms for result acceptance + interdisciplinary talent training + international technology transfer.	[18,44,57]

**Table 6:** Indicative staged outlook for aquatic-animal-disease POCT over the next 5–10 years.

Phase	Time Window	Core Milestones	Characteristics of Regional Progress	Potential Industry Impact	Representative References
Short term (1–3 years)	Already under way	Commercialization of dual- to five-target multiplex POCT products; gradual deployment of IoT strip readers and AI-assisted interpretation	Europe and the United States advance product validation, regulatory approval, and market entry; Asia-Pacific and Latin America expand application scale; underserved producing regions launch pilot programs	On-site confirmatory diagnosis compressed from “days” to “hours”; empirical and indiscriminate drug use hypothesized to decline, contingent on stewardship protocols (no included study yet quantifies the change)	[6,16,54,83,95]
Medium term (3–5 years)	R&D breakthroughs in progress	Cost reduction and mass production of microfluidic lab-on-a-chip devices; broad accessibility of one-pot CRISPR assays	Industrialized farming systems may achieve on-site automated testing; “testing + control” service packages roll out in developing countries; a preliminary, internationally harmonized framework for POCT performance evaluation is established	“Sample-in—result-out” becomes one of the mainstream on-site testing modes, with strengthened frontline disease-control capacity	[16,61,63]
Long term (5–10 years)	Frontier exploration	Mature AI aquaculture veterinary assistants; multimodal diagnostic closed loops; cold-chain-independent nanozyme/engineered-enzyme systems take shape	A global big-data network for aquatic animal health may emerge; international organizations establish POCT surveillance-data linkage mechanisms; cross-regional joint prevention and control may become routine	A precision disease-control system for global aquaculture takes preliminary shape, potentially underpinning food security and the sustainable development of the industry	[95,96,98,99,101]

Note: Expected reductions in indiscriminate antimicrobial use remain qualitative and are not yet supported by quantitative adoption data.

## Data Availability

No new data were created or analyzed in this study. Data sharing is not applicable to this article.

## Abbreviations

The following abbreviations are used in this manuscript:

AHPND	Acute Hepatopancreatic Necrosis Disease
AI	Artificial Intelligence
AMR	Antimicrobial Resistance
APHIS	Animal and Plant Health Inspection Service
CFU	Colony-Forming Unit
CRISPR	Clustered Regularly Interspaced Short Palindromic Repeats
DNA	Deoxyribonucleic Acid
EHP	Enterocytozoon Hepatopenaei
EQA	External Quality Assessment
FAM	6-Carboxyfluorescein
FIA	Fluorescent Immunochromatographic Assay
FRET	Fluorescence Resonance Energy Transfer
GAP	Global Action Plan on Antimicrobial Resistance
GCRV	Grass Carp Reovirus
GICA	Gold Immunochromatographic Assay
HNB	Hydroxynaphthol Blue
IHNV	Infectious Hematopoietic Necrosis Virus
IPNV	Infectious Pancreatic Necrosis Virus
ISAV	Infectious Salmon Anemia Virus
ISKNV	Infectious Spleen and Kidney Necrosis Virus
KHV	Koi Herpesvirus (Cyprinid Herpesvirus 3)
LAMP	Loop-Mediated Isothermal Amplification
LFD	Lateral-Flow Dipstick
LFIA	Lateral-Flow Immunoassay
MEMSs	Micro-Electromechanical Systems
MIP	Molecularly Imprinted Polymer
MRV	Mandarin Fish Ranavirus
MRL	Maximum Residue Limit
PAM	Protospacer Adjacent Motif
PDV	Pancreas Disease Virus
POCT	Point-of-Care Testing
RAS	Recirculating Aquaculture Systems
RAA	Recombinase-Aided Amplification
RPA	Recombinase Polymerase Amplification
RT	Reverse Transcription
SAV	Salmonid Alphavirus
SDDV	Scale Drop Disease Virus
SDG	Sustainable Development Goal
SERS	Surface-Enhanced Raman Scattering
SPE	Screen-Printed Electrode
SVCV	Spring Viraemia of Carp Virus
UCNP	Upconversion Nanoparticle
USD	United States Dollar
VHSV	Viral Hemorrhagic Septicemia Virus
WAHIS	World Animal Health Information System
WSSV	White Spot Syndrome Virus
AIE	Aggregation-Induced Emission
